# Refractory gastrocutaneous fistula treated by two-stage surgery: a case report

**DOI:** 10.1186/s40792-023-01788-4

**Published:** 2023-11-30

**Authors:** Yuji Kobayashi, Shusuke Yagi, Kazuhiko Yamada, Daiki Kato, Naoki Enomoto, Kyoko Nohara, Norihiro Kokudo

**Affiliations:** Department of Surgery, National Center for Global Health and Medicine, 1-21-1, Toyama, Shinjuku, Tokyo 162-8655 Japan

**Keywords:** Gastrocutaneous fistula, Enterocutaneous fistula, Open abdomen management, Gastrectomy

## Abstract

**Background:**

Gastrocutaneous fistulas are a rare complication of enterocutaneous fistulas and can be caused by intestinal injury, infection, and anastomotic leakage. They are typically treated conservatively or endoscopically; however, for large or difficult-to-treat gastrocutaneous fistulas, surgical intervention is required. Herein, we present a case of a huge gastrocutaneous fistula that was successfully treated with a two-stage surgery performed using open abdomen management.

**Case presentation:**

A 61-year-old man with a perforated gastric ulcer underwent omental filling as an emergency surgery. Post-operative leakage led the development of a 10-cm gastrocutaneous fistula. He was transferred to our hospital for the treatment of gastrocutaneous fistula. Furthermore, nutritional therapy was administered for dehydration, electrolyte abnormalities, metabolic acidosis, and acute kidney injury due to the high-output nature of the fistula. Moreover, owing to the intraperitoneal severe adhesion and poor nutritional status, two-stage surgery was planned. In the first stage, extensive dissection of the adhesions, distal gastrectomy reconstruction with Roux-en-Y anastomosis, and jejunostomy were performed. Furthermore, open abdomen management was conducted to check for the presence of unexpected complications due to extensive dissection of the adhesion and anastomotic leakage. Subsequently, in the second stage of the surgery, abdominal closure was performed on the 9th day after gastrectomy.

**Conclusion:**

Open abdomen management may be effective for huge gastrocutaneous fistulas with extensive adhesions that require surgical intervention.

## Background

Enterocutaneous fistulas are frequently caused by intestinal injury, infection, and anastomotic leakage after trauma and surgery, leading to malnutrition and wound infection, prolonged hospitalization, and high morbidity and mortality rates [1]. Among these fistulas, gastrocutanous fistulas are relatively rare. Notably, in a retrospective study of 277 patients with enterocutaneous fistulas and in a report of 135 patients in whom enterocutaneous fistulas were surgically repaired, only 1 and 2 cases of gastrocutaneous fistulas were reported, respectively [2, 3]. Gastrocutaneous fistulas are uncommon complications, accounting for 0.5–3.9% of gastric surgeries [4]. There have been several reports of gastrocutaneous fistula formation following removal of long-term gastrostomy tube placement [5, 6]; therefore, small gastrocutaneous fistulas are conservatively or endoscopically treated, and rarely require surgical intervention. Herein, we report a case of a huge gastrocutaneous fistula that required surgical intervention and was successfully treated with a two-stage surgery using open abdomen management.

## Case presentation

A 61-year-old man was admitted to a local hospital for abdominal pain and subsequently diagnosed with a perforated gastric ulcer and conservatively treated. However, owing to the subsequent worsening of his condition, an emergency surgery was performed. Omental filling of the perforated part of the gastric antrum and gastrostomy for gastric decompression were performed. Post-operative leakage subsequently occurred, and a gastrocutaneous fistula formed at the wound. He was transferred to our hospital for the treatment of gastrocutaneous fistula.

**Fig. 1:**
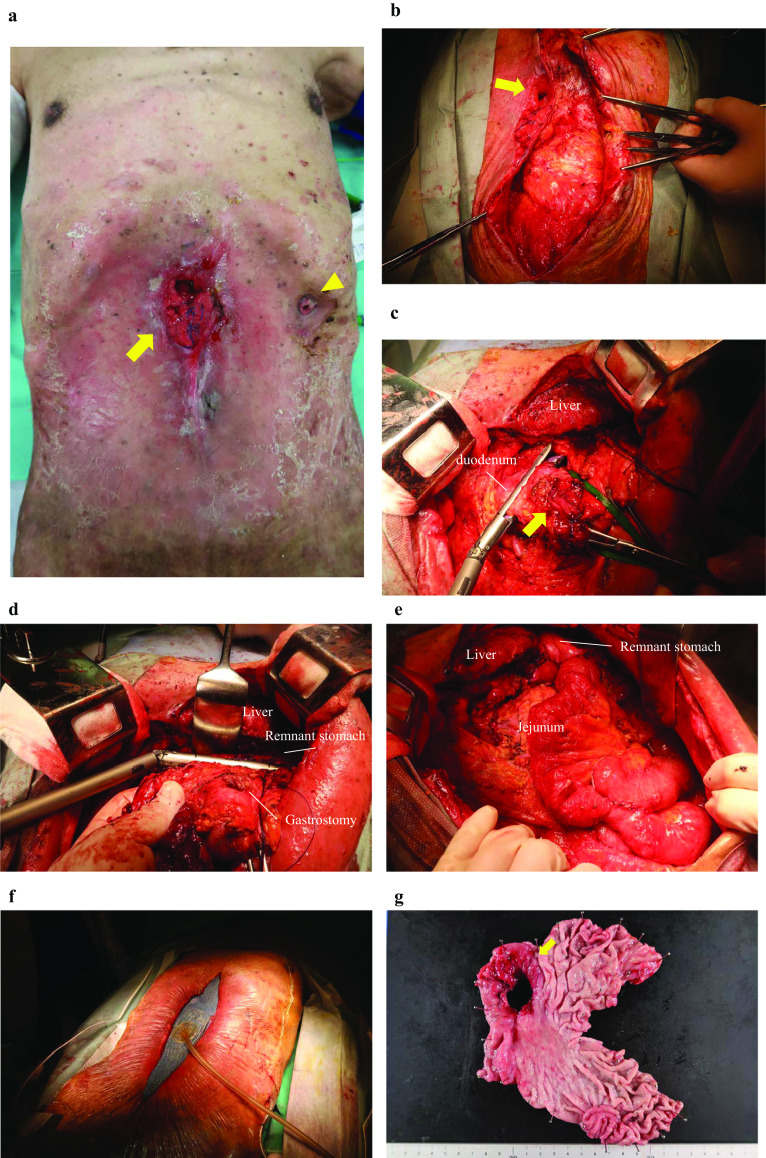
Surgical findings. A gastrocutaneous fistula (yellow arrow) (**a**) was observed in the epigastric region. A gastrostomy was noted on the left hypochondrium (yellow arrowhead) (**a**). Laparotomy with a midline abdomen was performed to avoid a gastrocutaneous fistula (yellow arrow). Severe intra-abdominal adhesions were observed (**b**). Resection of the stomach on the anal (**c**, yellow arrow; gastrocutaneous fistula) and oral sides (**d**) was performed. Roux-en-Y anastomosis was performed (**e**). Open abdomen management was performed with ABThera*™* (**f**). A fistula was found on the anterior side of the antrum of the stomach (**g**)

The gastrocutaneous fistula on his epigastric region was 10 cm in size. A gastrostomy was noted on his left hypochondrium (Fig. 1a). He had erythema with scales that was scattered throughout the body due to psoriasis. The huge gastrocutaneous fistula resulted in discharges of more than 1000 mL of gastric juice daily, indicating a high-output gastrocutaneous fistula. It subsequently caused dehydration, electrolyte abnormalities, metabolic acidosis, and acute kidney injury. Laboratory examination showed the following findings: hemoglobin, 10.4 g/dL; albumin, 2.7 g/dL; and creatinine, 1.37 mg/dL owing to the loss of gastric secretion. The test for *Helicobacter pylori* antibody was positive.

We attempted to improve the nutritional status of the patient by providing enteral nutrition for the fistula and central venous nutrition for the surgery. Enteral nutrition was performed by placing a feeding tube through the gastrocutaneous fistula into the jejunum using fluoroscopy.

The patient also had a skin disease and inflammatory induration around the fistula, which could have complicated abdominal closure. Therefore, the surgery was performed with preparation for open abdomen management.

**Fig. 2:**
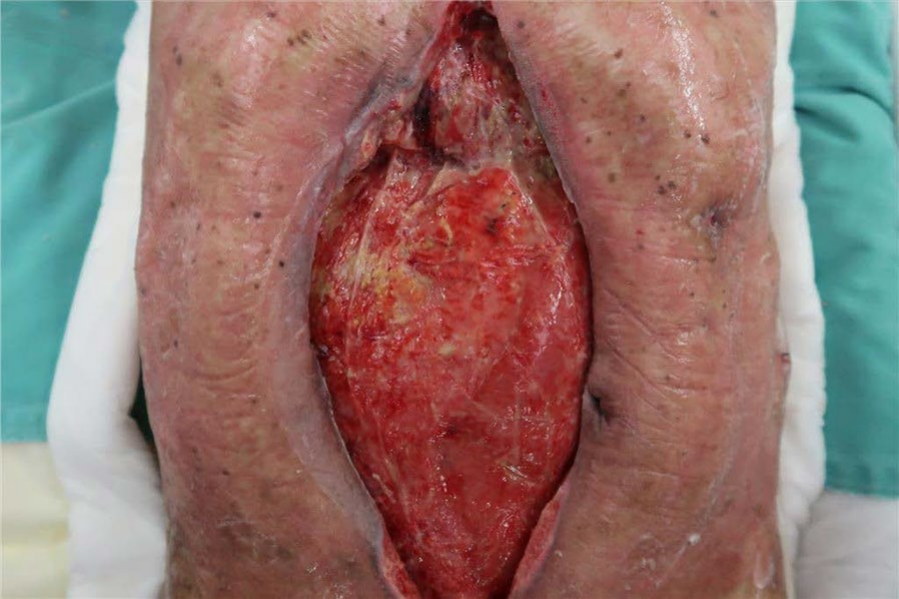
Open abdominal management. Replacement of the ABThera*™* to observe abdominal secondary injury

**Fig. 3:**
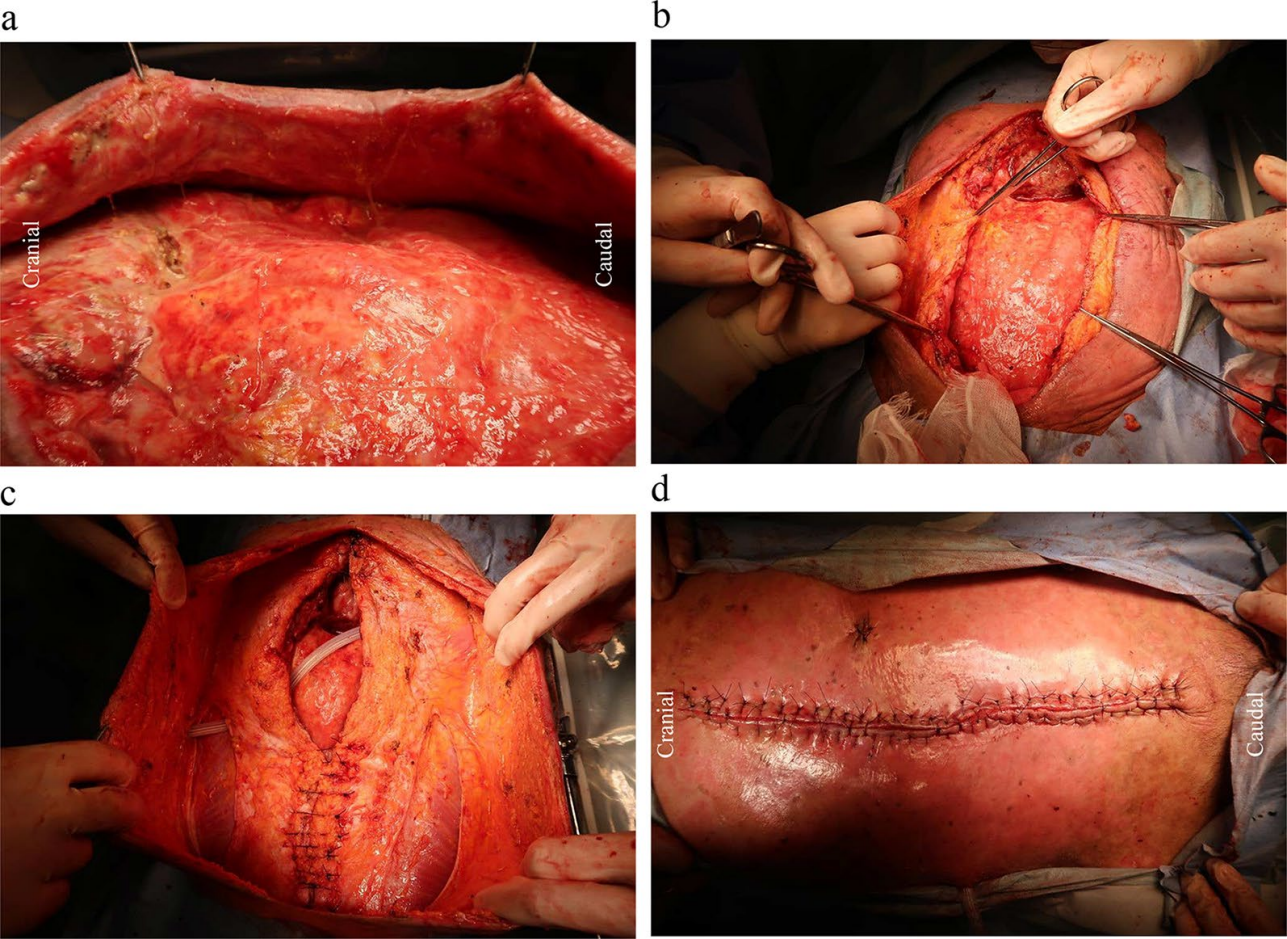
Secondary surgery. Abdominal organs were capsulized after open abdomen management (**a**). A fascia was expanded by undermine method and separated to abdominal closure (**b**, **c**). Abdominal closure by the secondary surgery (**d**)

Surgical findings revealed severe intra-abdominal adhesions. Further, distal gastrectomy reconstruction with Roux-en-Y anastomosis and jejunostomy were performed (Fig. 1b–e). As the preoperative condition of the patient was poor and extended adhesion peeling was performed, open abdomen management was performed to detect anastomotic leakage and unpredictable damage of adhesion peeling. Moreover, open abdomen management was performed using ABThera*™* (Fig. 1f). A fistula in the pylorus was found in the resected specimen. The fistula had no malignancy findings on pathology (Fig. 1g). The ABThera*™* device was replaced every 2 days for observation of gastrointestinal injury and anastomotic leakage (Fig. 2). Abdominal closure was performed 9 days after gastrectomy (Fig. 3).

The postoperative course was uneventful, and oral nutrition was resumed on postoperative day 7. He was discharged from the hospital without complications on postoperative day 80. Hospital stay was 128 days. At the time of discharge, his nutritional status improved, with albumin and creatinine levels of 3.5 g/dL and 1.79 mg/dL, respectively.

## Discussion

The treatment of enterocutaneous fistulas is because they frequently form skin erosions and ulcers around the fistula due to gastric secretion, activated pancreatic secretion, and bile-containing digestive secretion. Recently, devices and treatment methods for fistula closure have been developed. Gastrocutaneous fistulas can be almost completely treated by endoscopic management using clipping, suturing, and plugging methods [7]. Although surgical interventions are rarely performed as they are not as cost effective and safe as conservative treatment [8], they may be performed in some cases depending on the condition of the fistula, because they are more effective in treating fistulas than conservative treatment. Haack et al. recommended spontaneous closure of fistula of unfavorable origins, including the stomach, duodenum, proximal jejunum, and ileum [9]. Moreover, unfavorable fistulas were categorized on the basis of fistula character and environment. In addition, fistula output, nutrition status, transferrin levels, and sepsis were the factors that prevented spontaneous closure [9]. In the present case, the patient was malnourished and had a fistula in the stomach with a large enteric defect, budding mucosa, and high-output leakage, posing difficulty in performing spontaneous closure. Therefore, surgical therapy was considered favorable.

Open abdomen management has been used as a strategy for damage control surgery for severe trauma. Recently, it has also been used in nontraumatic cases [10, 11].

Open abdomen management aims to control septic peritonitis, abdominal fluid, and abdominal compartment syndrome to facilitate repeated abdominal exploration. This management is useful for the early detection of postoperative complications in the abdomen, including anastomotic leakage and infection, which can cause an enterocutaneous fistula. We could observe the intraperitoneal space every two days by the dressing kit of ABThera™ changing. A second look was effective to observe accidental secondary intestinal injuries. In our case, repeated abdominal exploration was effective for observing secondary intestinal injuries following adhesion peeling due to severe abdominal adhesions. Repeated abdominal exploration revealed no injuries. Open abdomen management was useful in observing secondary injuries and anastomotic leakage during both traumatic and nontraumatic surgeries where adhesion peeling was extensive or risk of anastomosis was high.

## Conclusions

Open abdomen management may be an effective treatment option for huge gastrocutaneous fistulas with extensive adhesions that require surgical intervention.

## Data Availability

The data set supporting the conclusions of this article is available in the Springer Open.
